# Frequency of visits to health facilities and HIV services offered to men, Malawi

**DOI:** 10.2471/BLT.20.278994

**Published:** 2021-06-29

**Authors:** Kathryn Dovel, Kelvin Balakasi, Sundeep Gupta, Misheck Mphande, Isabella Robson, Shaukat Khan, Alemayehu Amberbir, Christian Stilson, Joep J van Oosterhout, Naoko Doi, Brooke E Nichols

**Affiliations:** a Division of Infectious Diseases, David Geffen School of Medicine, University of California Los Angeles, 10833 Le Conte Ave, Los Angeles, CA 90095, United States of America (USA).; b Partners in Hope, Lilongwe, Malawi.; c Clinton Health Access Initiative, Boston, USA.; d Department of Global Health, School of Public Health, Boston University, Boston, USA.

## Abstract

**Objective:**

To determine how often men in Malawi attend health facilities and if testing for human immunodeficiency virus (HIV) is offered during facility visits.

**Methods:**

We conducted a cross-sectional, community-representative survey of men (15–64 years) from 36 villages in Malawi. We excluded men who ever tested HIV-positive. Primary outcomes were: health facility visits in the past 12 months (for their own health (client visit) or to support the health services of others (guardian visit)); being offered HIV testing during facility visits; and being tested that same day. We disaggregated all results by HIV testing history: tested ≤ 12 months ago, or in need of testing (never tested or tested > 12 months before).

**Findings:**

We included 1116 men in the analysis. Mean age was 34 years (standard deviation: 13.2) and 55% (617/1116) of men needed HIV testing. Regarding facility visits, 82% (920/1116) of all men and 70% (429/617) of men in need of testing made at least one facility visit in the past 12 months. Men made a total of 1973 visits (mean two visits): 39% (765/1973) were as guardians and 84% (1657/1973) were to outpatient departments. Among men needing HIV testing, only 7% (30/429) were offered testing during any visit. The most common reason for not testing was not being offered services (37%; 179/487).

**Conclusion:**

Men in Malawi attend health facilities regularly, but few of those in need of HIV testing are offered testing services. Health screening services should capitalize on men’s routine visits to outpatient departments as clients and guardians.

## Résumé

**Objectif:**

Déterminer la fréquence à laquelle les hommes se rendent dans des établissements de soins au Malawi, et savoir si un dépistage du virus de l'immunodéficience humaine (VIH) est proposé lors de ces visites.

**Méthodes:**

Nous avons mené une enquête transversale représentative de la communauté auprès d'hommes (15–64 ans) résidant dans 36 villages malawites. Nous n'y avons pas inclus les hommes testés positifs au VIH. Les critères primaires étaient les suivants: visites dans des établissements de soins au cours des 12 derniers mois pour leur propre santé (visite en qualité de patient) ou pour la santé d'autrui (visite en qualité de tuteur); proposition de dépistage du VIH lors de ces visites; et enfin, test effectué le jour même. Nous avons ensuite ventilé l'ensemble des résultats en fonction des antécédents de dépistage du VIH: test réalisé ≤ 12 mois auparavant, ou test nécessaire (car jamais réalisé ou réalisé > 12 mois auparavant).

**Résultats:**

Notre analyse s'est intéressée à 1116 hommes âgés de 34 ans en moyenne (écart type: 13,2), dont 55% (617/1116) avaient besoin d'un dépistage du VIH. Concernant les visites dans des établissements de soins, 82% (920/1116) des hommes dans leur globalité et 70% (429/617) des hommes ayant besoin d'un dépistage ont effectué au moins une visite au cours des 12 derniers mois. Le groupe étudié s'est rendu 1973 fois dans de tels établissements (ce qui équivaut à deux visites en moyenne): 39% (765/1973) sont venus en qualité de tuteur, et 84% (1657/1973) se sont adressés à des services de consultation externes. Parmi ceux qui avaient besoin d'un dépistage du VIH, seulement 7% (30/429) se sont vu proposer un test lors de l'une de leurs visites. Cette absence de test est principalement due à l'indisponibilité du service (37%; 179/487).

**Conclusion:**

Au Malawi, les hommes se rendent régulièrement dans des établissements de soins, mais rares sont ceux qui se voient proposer un test VIH malgré le besoin. Les services de dépistage devraient miser sur ces visites de routine dans des cliniques externes, que les hommes soient tuteurs ou patients.

## Resumen

**Objetivo:**

Determinar la frecuencia con la que los hombres de Malawi acuden a los centros de salud y si se ofrecen pruebas del virus de la inmunodeficiencia humana (VIH) durante las visitas a dichos centros.

**Métodos:**

Se realizó una encuesta transversal, representativa de la comunidad, de hombres (de 15 a 64 años) de 36 pueblos de Malawi. Se excluyeron los hombres que alguna vez dieron positivo en la prueba del VIH. Los resultados primarios fueron: visitas a centros de salud en los últimos 12 meses (para su propia salud [visita del paciente] o para apoyar los servicios de salud de otros [visita de cuidador]); que se les ofreciera la prueba del VIH durante las visitas a los centros; y que se les hiciera la prueba ese mismo día. Desglosamos todos los resultados según el historial de pruebas del VIH: se hicieron la prueba hace ≤12 meses, o necesitan la prueba (nunca se hicieron la prueba o se hicieron la prueba hace >12 meses antes).

**Resultados:**

Se incluyeron 1116 hombres en el análisis. La edad media fue de 34 años (desviación estándar: 13,2) y el 55 % (617/1116) de los hombres necesitaban la prueba del VIH. En cuanto a las visitas a los centros, el 82 % (920/1116) de todos los hombres y el 70 % (429/617) de los hombres que necesitaban la prueba realizaron al menos una visita a un centro en los últimos 12 meses. Hubo un total de 1973 visitas (con una media de dos visitas): el 39 % (765/1973) fueron como cuidadores y el 84 % (1657/1973) fueron a departamentos ambulatorios. Entre los hombres que necesitaban someterse a la prueba del VIH, solo al 7% (30/429) se les ofreció la prueba durante cualquier visita. La razón más común para no hacerse la prueba fue que no se les ofrecieron servicios (un 37 %; 179/487).

**Conclusión:**

Los hombres de Malawi acuden a los centros de salud con regularidad, pero a pocos de los que necesitan someterse a la prueba del VIH se les ofrecen los servicios. Los servicios de detección sanitaria deberían aprovechar las visitas rutinarias de los hombres a los departamentos ambulatorios como pacientes y cuidadores.

## ملخص

**الغرض:**

تحديد عدد المرات التي يذهب فيها الرجال في ملاوي إلى المرافق الصحية، وما إذا كان يُعرض عليهم إجراء اختبار فيروس نقص المناعة البشرية (HIV) أثناء زيارات المرفق.

**الطريقة:**

أجرينا مسحًا مقطعيًا تمثيليًا للمجتمع من الرجال (في سن 15 إلى 64 عامًا) من 36 قرية في ملاوي. وقمنا باستبعاد الرجال الذين ثبتت إصابتهم بفيروس نقص المناعة البشرية (HIV). وكانت النتائج الأولية كالتالي: زيارات المرافق الصحية خلال الاثني عشر شهرًا الماضية (من أجل صحتهم (زيارة كمريض) أو لدعم الخدمات الصحية للآخرين (زيارة كولي للأمر))؛ عرض إجراء اختبار فيروس نقص المناعة البشرية أثناء زيارات المرفق؛ وإجراء الاختبار في نفس هذا اليوم. قمنا بتصنيف جميع النتائج حسب تاريخ اختبار فيروس نقص المناعة البشرية: ما تم إجراء الاختبار عليه منذ 12 شهرًا أو أقل، أو ما هو في حاجة للاختبار (لم يتم اختباره أو تم اختباره منذ أكثر من 12 شهرًا).

**النتائج:**

قمنا بتضمين 1116 رجلًا في التحليل. كان متوسط ​​العمر 34 عامًا (الانحراف المعياري: 13.2) واحتاج 55% (617/1116) من الرجال إلى إجراء اختبار فيروس نقص المناعة البشرية. فيما يتعلق بزيارات المرافق، فإن 82% (920/1116) من جميع الرجال، و70% (429/617) من الرجال الذين يحتاجون إلى الاختبار، قاموا بزيارة واحدة على الأقل للمنشأة خلال الاثني عشر شهرًا الماضية. قام الرجال بإجمالي عدد 1973 زيارة (بمتوسط زيارتين): 39% (765/1973) كانت من أولياء الأمور، و84% (1657/1973) كانت إلى العيادات الخارجية. من بين الرجال الذين يحتاجون إلى إجراء اختبار فيروس نقص المناعة البشرية، تم فقط على 7% منهم (30/429) عرض إجراء الاختبار خلال أية زيارة. كان السبب الأكثر شيوعًا لعدم إجراء الاختبار هو عدم عرض الخدمات (37%؛ 179/487).

**الاستنتاج:**

يذهب الرجال في ملاوي إلى المرافق الصحية بانتظام، لكن القليلين من الذين يحتاجون إلى إجراء اختبار فيروس نقص المناعة البشرية، يُعرض عليهم خدمات إجراء الاختبار. يجب أن تستفيد خدمات الفحص الصحي من الزيارات الروتينية للرجال إلى العيادات الخارجية بصفتهم مرضى وأولياء للأمر. ****

## 摘要

**目的:**

旨在确定马拉维男子前往卫生机构就诊的频率，以及就诊期间是否接受人体免疫缺陷病毒 (HIV) 检测。

**方法:**

我们面向马拉维 36 个村庄的男子（15 至 64 岁）开展了一项具有社区代表性的横断面调查。我们排除了曾经检测过 HIV，且检测结果呈阳性的男子。调查结果主要包括：在过去 12 个月前往过卫生机构就诊【为了自己的健康（本人就诊）或为了照顾他人健康（作为监护人代为就诊）】；就诊期间接受过 HIV 检测；就诊和检测是在同一天。我们按照 HIV 检测情况对所有结果进行分类：12 个月以内检测过，或需要检测（从未检测或超过 12 个月以前检测过）。

**结果:**

我们在分析中纳入了 1,116 名男子。这些人的平均年龄是 34 岁（标准差：13.2），且 55% (617/1116) 的男子需要进行 HIV 检测。关于就诊，所有男子中有 82% (920/1116) 且需要检测的男子中有 70% (429/617) 在过去 12 个月内就诊过至少一次。男子就诊的次数共计 1973 次（平均两次）: 39% (765/1973) 是以监护人的身份代为就诊，而 84% (1657/1973) 则前往门诊亲自就诊。在需要接受 HIV 检测的男子中，只有 7% (30/429) 在任意一次就诊期间均接受了检测。不检测的最常见原因是不提供这项服务 (37%;179/487)。

**结论:**

马拉维的男子经常前往卫生机构就诊，但需要 HIV 检测的男子很少能够接受这项检测服务。应该充分利用男子本人和作为监护人定期前往门诊就诊的机会来提供健康筛查服务。

## Резюме

**Цель:**

Определить, как часто мужчины в Малави посещают медицинские учреждения и предлагают ли им пройти тестирование на вирус иммунодефицита человека (ВИЧ) во время посещения медицинских учреждений.

**Методы:**

Мы провели поперечный репрезентативный опрос среди мужчин (от 15 до 64 лет) из 36 деревень Малави. Мы исключили мужчин, которые ранее имели положительный результат тестирования на ВИЧ-инфекцию. Первичными данными для анализа были посещения медицинского учреждения за последние 12 месяцев (в связи со своим собственным здоровьем (посещение в роли пациента) или для оказания поддержки в получении услуг здравоохранения другим лицам (посещение в роли опекуна)), предложение пройти тестирование на ВИЧ во время посещения медицинского учреждения, а также прохождение тестирования в тот же день. Мы разделили все результаты по анамнезу тестирования на ВИЧ: проходили тестирование ≤ 12 месяцев назад или нуждаются в тестировании (не проходили тестирования никогда или > 12 месяцев назад).

**Результаты:**

В анализ были включены 1116 мужчин. Средний возраст составлял 34 года (стандартное отклонение: 13,2); 55% (617/1116) мужчин нуждались в тестировании на ВИЧ. Что касается посещений медицинских учреждений, 82% (920/1116) всех мужчин и 70% (429/617) мужчин, которые нуждались в тестировании, посетили медицинское учреждение по крайней мере один раз за последние 12 месяцев. Мужчины совершили в общей сложности 1973 посещения (в среднем два посещения): 39% (765/1973) были опекунами и 84% (1657/1973) посещали амбулаторные отделения. Среди мужчин, которые нуждались в тестировании на ВИЧ, только 7% (30/429) было предложено пройти тестирование во время посещения. Наиболее частой причиной непрохождения тестирования был тот факт, что такую услугу просто не предлагали (37%; 179/487).

**Вывод:**

Мужчины в Малави регулярно посещают медицинские учреждения, но немногим мужчинам, которые нуждаются в тестировании на ВИЧ, предлагают такую услугу. Медицинские службы должны наилучшим образом воспользоваться фактом регулярного посещения мужчинами амбулаторных отделений как в качестве пациентов, так и в качестве опекунов.

## Introduction

Men throughout sub-Saharan Africa have a lower life expectancy than women.^1^ One contributing factor is men’s underrepresentation in early disease detection, for example, timely diagnosis of life-threatening conditions such as tuberculosis, human immunodeficiency virus (HIV) infection, and hypertension.^2^^–^^5^ Low uptake of routine screening services is concerning because delayed diagnosis can result in advanced stages of illness and increased risk of onward HIV or tuberculosis transmission. Effective strategies are needed to reach men with routine screening services. 

Facility-based interventions for routine screening services can increase men’s engagement in care and may be more scalable and sustainable than community-based approaches, particularly in resource-constrained settings where human resources are limited.^6^^,^^7^ Yet, most interventions for men prioritize community-based approaches,^8^^–^^10^ in part due to assumptions that men do not attend health facilities and intentionally avoid health facilities altogether.^11^^,^^12^ A common belief is that community services are required to find so-called missing men who otherwise cannot be reached.^10^^,^^13^ Little is known, however, about how often men actually attend health facilities, either as clients or in support of the health care of others (i.e. as guardians). Facility-based strategies during routine facility visits may be a feasible way to reach most men if men frequent health facilities.

While men in sub-Saharan Africa are less likely than women to access testing and treatment services for HIV and tuberculosis,^14^^,^^15^ these services likely comprise only a minority of men’s facility visits. Men may attend outpatient departments for acute care, or attend a variety of other departments as guardians to aid others seeking care, such as spouses or children. A study in Malawi found that 22% (90/401) of rural men had accessed health services for their own health in the previous 2 months.^16^ However, the proportion of men attending health facilities over time is still unclear because findings were limited to visits for men’s own health care (not as guardians) and only visits within the previous 2 months.

It is also unclear if men who attend health facilities for reasons other than HIV and tuberculosis services are offered screening services (such as HIV, tuberculosis or hypertension screening) during their facility visits. Research suggests that screening coverage during routine care may be low for a range of diseases,^17^^,^^18^ especially among individuals attending outpatient departments that are often busy and overburdened. Low screening coverage could indicate missed opportunities to engage men already attending health facilities. Examining the frequency with which men attend health facilities, and whether screening services are offered when they do attend, is important to understand the potential role of facility-based interventions for men.

We used HIV testing as a case study to explore the potential role of facility-based screening strategies for men. We focused on HIV testing because Malawian national guidelines indicate that sexually active men should be tested for HIV every 12 months, regardless of symptoms or age.^19^ HIV testing is also decentralized and widely available in nearly every health facility in Malawi. We aimed to determine the frequency with which men in Malawi attend health facilities and the HIV services offered to them when they do attend.

## Methods

### Setting

In Malawi, an estimated 11% of men living with HIV are unaware of their HIV status compared with 6% of women.^20^ According to national guidelines, facility-based testing should be offered through provider-initiated testing and counselling, whereby providers offer HIV testing services to clients in need of testing (defined as those who have never tested positive and did not test <12 months ago).^19^ Implementation of provider-initiated testing and counselling varies widely by facility department, with nearly 100% coverage in antenatal clinics^21^ and less than 15% testing coverage in outpatient departments.^22^^,^^23^

### Design and participants 

We conducted a cross-sectional, community-representative survey of men in 36 villages in the central and southern regions of Malawi. We used a multistage sampling design to select study villages and potential respondents. We purposively selected two districts in central and southern regions (Lilongwe and Chikwawa) and three mid-size health facilities per district (six facilities in total). We then randomly selected six villages within each facility’s catchment area by using a computer-generated sequence of random numbers (36 villages in total), and used household census listings to randomly select men within each village. Villages were a mean of 5.14 km (standard deviation, SD: 3.46 km) from facilities, had a mean of 72.8 (SD: 27.7) households per village, and the main occupations were farming, fishing and informal employment (further details available in the data repository).^24^

Eligibility criteria for inclusion in the survey were: (i) age 15–64 years; (ii) current resident of the participating village; and (iii) spent > 15 nights in the village in the previous 30 days. We excluded men who did not meet eligibility criteria from the final household listing before randomization. For this paper, we also excluded men who self-reported ever testing HIV-positive. We stratified the random selection of men by village (about 45 men per village, although some villages had fewer than 45 men due to small village size) and age category: young men (15–24 years), middle-aged men (25–39 years) and older men (≥ 40 years). We used computerized random number generation to select strata.

### Data collection and measurement

Male research assistants recruited randomly selected individuals with the assistance of community health workers and village chiefs. We categorized individuals as not found after three failed tracing attempts. We conducted surveys wherever convenient for respondents, which was usually at the respondent’s home, the village chief’s residence (a typical gathering place in villages) or the respondent’s place of work. 

We collected the following data: (i) demographic data, such as age, marital status and education; (ii) self-reported HIV testing history, defined as tested recently (testing HIV-negative in the past 12 months) or in need of testing (never tested positive or tested > 12 months ago), as per national guidelines;^25^ (iii) number of health facility visits made in the previous 24 months; (iv) services received during the last four health facility visits, including who received the services (i.e. client or guardian visit type); and (v) if HIV testing was offered and received during each facility visit. We defined client visits as any visit where the primary service received was for the respondent’s own health. We defined guardian visits as any visit where the primary service was received by another person and the respondent attended the facility to support that person’s use of health services – we did not categorize providing transportation and immediately leaving the facility as a guardian visit. We defined being offered HIV testing as being told about HIV testing by a health-care worker at the facility on the same day as a facility visit, and actual HIV testing as completing an HIV test the same day as a facility visit. We conducted surveys in the local language (Chichewa) and they lasted about 55 minutes on average.

### Analysis and sample size

Our primary outcome was health facility attendance in the past 12 months – we excluded any health visits made more than 12 months before the survey. Secondary outcomes included being offered HIV testing during facility visits in the past 12 months and completing an HIV test at any facility visit in the same period. Our study included 1116 men (HIV-negative or with an unknown HIV status), which meant that it had had over 80% power to estimate population-level frequency of health-facility visits within the past 12 months, assuming 5% precision, 0.05 level of confidence and about 10 000 men and male adolescents in each facility catchment area.

We used descriptive statistics to examine how often men attend health facilities and the reason for facility visits (client or guardian), disaggregated by history of HIV testing (tested in the past 12 months or in need of testing). We conducted sensitivity analyses using assigned weights to account for variation in village size. The results from sensitivity analyses did not change findings and are available in the data repository.^24^ No data were missing.

### Ethical considerations

The National Health Sciences Review Committee of Malawi (number 2338) and the University of California Los Angeles Institutional Review Board (number 20–001606) approved study activities. All eligible individuals completed a written informed consent form immediately following screening procedures. For individuals between 15 and 18 years of age, guardians provided written consents.

## Results

**Fig. 1:**
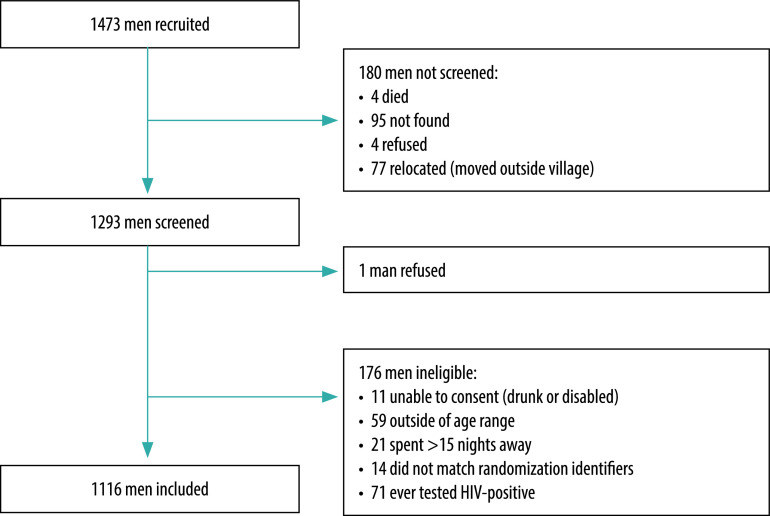
Flowchart for inclusion of men in the study, Malawi, 2019

We recruited 1473 men between 15 August and 18 October 2019. Of these men, we screened 1293 (88%) men for eligibility – we were unable to screen 180 (12%) men (Fig. 1). Of the 1293 men screened, 1117 (86%) were eligible for inclusion in the study. One man declined to participate in the study and thus we included 1116 men in the analysis (Fig. 1).

Table 1 shows the demographic data of the respondents according to history of HIV testing. The mean age of men was 34 years (SD: 13.2), 84% (941/1116) felt healthy and 82% (910/1116) were married or living with a partner. Most men (69%; 767/1116) had worked in the previous 30 days and 27% (299/1116) had spent three nights or more away from their home in the same period.

**Table 1:** Characteristics of men participating in the community survey, by HIV testing history, Malawi, 2019

Characteristic	All men (*n* = 1116)	Men recently tested (*n* = 499)	Men in need of testing^a^ (*n* = 617)	*P* ^b^
**Demographic data**				
Age in years, mean (SD)	34 (13.2)	35 (12.2)	34 (14.0)	0.16
Education completed in years, mean (SD)	6 (3.4)	6 (3.5)	6 (3.3)	< 0.001
Household wealth quintile, no. (%)				0.14
Poor	372 (33)	152 (30)	220 (36)	
Middle income	372 (33)	179 (36)	193 (31)	
Wealthy	372 (33)	168 (34)	204 (33)	
Number of children living in household, mean (SD)	3 (2.4)	3 (2.3)	3 (2.4)	< 0.001
Self-rated health (good or very good), no. (%)	941 (84)	415 (83)	526 (85)	0.341
**School, work and travel, no. (%)**				
Currently attending school (secondary or above)	49 (4)	28 (6)	21 (3)	0.07
Worked for pay in previous month (formal or informal)	767 (69)	365 (73)	402 (65)	< 0.001
Slept ≥ 3 nights away from home in past 30 days	299 (27)	149 (30)	150 (24)	0.03
**Sexual partnerships**				
Married or living together, no. (%)	910 (82)	443 (89)	467 (76)	< 0.001
Length of current relationship, in years, mean (SD)	13 (10)	12 (10)	14 (11)	0.05
**Sexual risk behaviour, no. (%)**				
Two or more sexual partner in past 12 months	345 (31)	174 (35)	171 (28)	0.01
Had sex with a non-married or live-in partner without using a condom	198 (18)	91 (18)	107 (17)	0.70
Partner known to be HIV-positive	14 (1)	8 (2)	6 (1)	0.54
**HIV testing, no. (%)**				
Tested ≤ 12 months before	499 (45)	499 (100)	0 (0)	NA
In need of HIV testing	617 (55)	0 (0)	617 (100)	NA
Never tested	294 (26)	NA	294 (48)	
Tested > 12 months ago	323 (29)	NA	323 (52)	

HIV: human immunodeficiency virus; NA: not applicable; SD: standard deviation.

^a^ Defined as never been tested or tested more than 12 months before.

^b^ χ^2^ test, *t*-test or Kruskal–Wallis test.

Participants commonly reported risky sexual behaviour, with 31% (345/1116) reporting having two sexual partners or more in the past 12 months and 18% (198/1116) reporting not using a condom with at least one non-married or live-in partner in the same period. At the time of the study, 55% (617/1116) of the men needed HIV testing (defined as never tested or tested > 12 months ago) – 26% (294/1116) had never been tested.

**Fig. 2:**
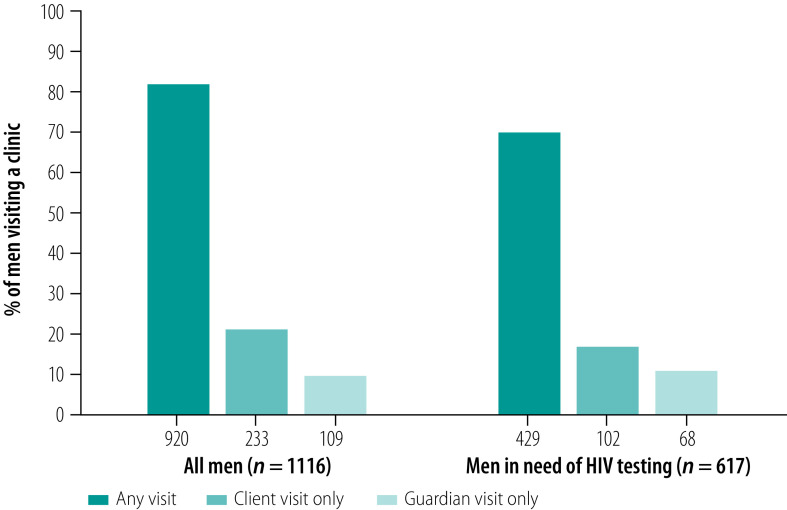
Men’s attendance at a health facility in the past 12 months, by visit type, Malawi, 2019

Of the total number of men included in the study, 82% (920/1116) had made at least one facility visit as either a client or guardian in the past 12 months, and 70% (429/617) of men in need of HIV testing had made at least one facility visit (Fig. 2). Secondary analyses show a similar frequency of facility visits among men who had never been tested (data repository).^24^ We found 10% (109/1116) of all men and 11% (68/617) of men in need of HIV testing only attended facilities as a guardian (i.e. made no visits as a client).

HIV: human immunodeficiency virus.

Note: Any visit also includes men who made both client and guardian visits during the 12 months.

A total of 1973 health facility visits were made by 920 men, with a mean two visits (SD 1–3) per participant, in the past 12 months (Table 2). Of all health visits made, 39% (765/1973) were made as guardians. Among men in need of testing, 48% (373/771) of visits were guardian visits. Most visits were to outpatient department clinics (84%; 1653/1973), regardless of visit type (client or guardian). Very few men attended a facility to support women’s reproductive health services or preventive services for children younger than 5 years. Among guardian visits, 42% (318/765) of men were accompanying children, 26% (199/765) were accompanying friends and 24% (182/765) were accompanying their sexual partner. Men in need of testing made significantly fewer facility visits, were more likely to attend a facility as a guardian (not a client), and were more likely to attend the outpatient department than men who had recently been tested for HIV (*P* < 0.001 for all visit characteristics). Men in need of testing were also significantly less likely than recently tested men to accompany their sexual partners during guardian visits (*P* < 0.001) – those in need of testing primarily accompanied children, friends or other relatives. 

**Table 2:** Facility visits made by men in the past 12 months, by visit type and HIV testing history, Malawi, 2019

Type of visit	All men	Men recently tested	Men in need of testing^a^	*P* ^b^
**All visits, *n***	1973	1202	771	
Among men who made any visit in the past 12-months, median no. of all visits (IQR)	2.0 (1.0–3.0)	2.0 (2.0–3.0)	2.0 (1.0–2.0)	< 0.001
Visit type, no. (%)				< 0.001
Guardian visit	765 (39)	392 (33)	373 (48)	
Client visit	1208 (61)	810 (67)	398 (52)	
**Guardian visits, *n***	765	392	373	
Among men who made any visit in the previous 12 months, median no. of visits in that time (IQR)	2.0 (1.0–2.0)	2.0 (1.0–3.0)	2.0 (1.0–2.0)	< 0.001
Client relationship with male, no. (%)				< 0.001
Child	318 (42)	155 (40)	163 (44)	
Partner	182 (24)	122 (31)	60 (16)	
Friend or other relative	199 (26)	84 (21)	115 (31)	
Other	66 (9)	31 (8)	35 (9)	
Main department where services were accessed, no. (%)				< 0.001
Outpatient	646 (84)	310 (79)	336 (90)	
HIV testing and counselling^c^	3 (< 1)	2 (1)	1 (< 1)	
Antiretroviral therapy	8 (1)	6 (1)	2 (< 1)	
Female reproductive health^d^	89 (12)	64 (16)	25 (7)	
Services for children younger than 5 years	5 (1)	4 (1)	1 (< 1)	
Dentist	3 (< 1)	1 (< 1)	2 (< 1)	
Other	11 (1)	5 (1)	6 (2)	
**Client visits, *n***	1208	810	398	
Among men who made any visit in the past 12 months, median no. of client visits in that time (IQR)	2.0 (1.0–3.0)	2.0 (2.0–3.0)	1.0 (1.0–2.0)	< 0.001
Main department services were accessed, no. (%)				< 0.001
Outpatient department	1007 (83)	623 (77)	384 (96)	
HIV testing and counselling^c^	171 (14)	169 (21)	2 (< 1)	
Female reproductive health^d^	2 (< 1)	2 (< 1)	0 (0)	
Dentist	17 (1)	7 (1)	10 (3)	
Other	11 (1)	9 (1)	2 (< 1)	

HIV: human immunodeficiency virus; IQR: interquartile range.

^a^ Defined as never been tested or tested more than 12 months before.

^b^ χ^2^ test, *t*-test or Kruskal–Wallis test.

^c^ Facility visits where HIV testing and counselling was the main reason for men attending the facility.

^d^ For antenatal care, family planning or delivery.

Table 3 shows HIV testing services offered and used among men who attended a health facility. Among all men who attended a facility in the past 12 months, 25% (233/920) were offered provider-initiated HIV testing and counselling during at least one facility visit and 48% (441/920) were tested for HIV. A higher proportion were tested than offered testing because some men only attended facilities for HIV testing services or actively sought out HIV testing during their facility visit (without being prompted by a health-care worker). When excluding men who only attended facilities for HIV testing services (defined as only attended the HIV testing and counselling department, 143 men), 38% (298/777) of men were tested for HIV during a facility visit outside an HIV testing and counselling department.

**Table 3:** Offer and use of HIV testing among men visiting health facilities in the past 12 months, by HIV testing history, Malawi, 2019

Variable	All men, no. (%) (*n* = 920)	Men recently tested, no. (%) (*n* = 491)	Men in need of testing,^a^ no. (%) (*n* = 429)	*P* ^b^
**Testing services**				
Offered provider-initiated testing and counselling at least once	233 (25)	203 (41)	30 (7)	< 0.001
Tested for HIV at least once	441 (48)	442 (90)	0 (0)	< 0.001
Tested for HIV, excluding at visits to HIV testing and counselling department	298/777 (38)	296/344 (86)	0 (0)	< 0.001
**Reason for not testing during most recent visit (*n* = 484)** ^c^				
Not offered testing	NA	NA	179 (37)	NA
Perceived low risk of infection	NA	NA	113 (23)	NA
Not ready to test	NA	NA	81 (17)	NA
Other	NA	NA	84 (17)	NA
Requires too much time	NA	NA	23 (5)	NA
Lack of privacy	NA	NA	4 (1)	NA

HIV: human immunodeficiency virus; NA: not applicable.

^a^ Defined as never been tested or tested more than 12 months before.

^b^ χ^2^ test, *t*-test or Kruskal–Wallis test.

^c^ Respondents were allowed to give more than one reason.

**Fig. 3:**
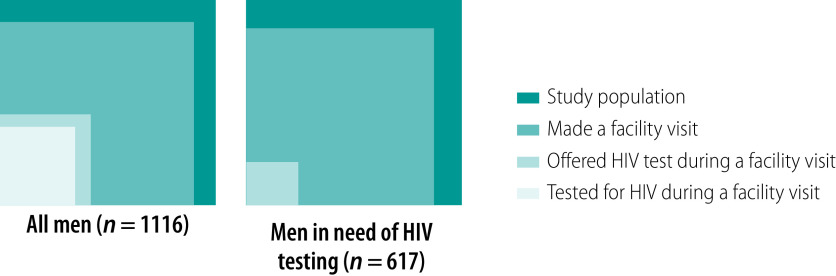
Proportion of men visiting a health facility in the past 12 months, offered HIV testing and accepted HIV testing, Malawi, 2019

Fig. 3 depicts missed opportunities for reaching men with HIV testing. Among all men, the biggest gap in the cascade for facility-based testing was being offered HIV testing. While 82% (920/1116) of men had made at least one facility visit in the past 12 months, only 25% (233/920) were offered provider-initiated HIV testing and counselling (Fig. 3). Of those offered these services, 198 (85%) accepted them and were tested for HIV that same day. Among men in need of testing, 70% (429/617) had made at least one facility visit in the past 12 months, but only 30 (7%) were offered provider-initiated HIV testing and counselling, none of whom accepted testing.

HIV: human immunodeficiency virus.

Note: Absolute numbers are presented in Table 3.

The most commonly reported reasons for not testing during their most recent facility visit were: not offered testing (37%; 179/484); perceived low risk of infection (23%; 113/484); and not ready to test (17%; 81/484). Reasons reported were similar for client and guardian visits (data repository).^24^ Among men making guardian visits, only 13% (35/268) were not willing to test because of lack of privacy because they were accompanying someone else.

## Discussion 

Few studies have examined men’s general health-seeking behaviour in sub-Saharan Africa, or the role of men as guardians. Using data from a community-representative survey of 36 villages in Malawi, we show that more than three quarters of men with an HIV-negative or unknown HIV status had attended a health facility in the past 12 months. Over 80% of all visits to health facilities were to an outpatient department for acute, curative services; 39% of all visits were as a guardian to support the health care of others. Among men in need of HIV testing, 70% had attended a health facility in the past 12 months. Over 90% of their facility visits were to an outpatient department and 48% of visits were as guardians. 

Our findings challenge the common belief that men do not attend health facilities, which has important implications for programmes targeting men. If facility-based services can be scaled up, most men may be reached during routine outpatient department visits. Targeted community-based strategies could then focus on the small proportion of high-risk men who do not regularly attend facilities, which could improve the efficiency and overall sustainability of HIV and other health screening programmes. However, facility-based services should capitalize on every visit made by men. Men in our study made a median of two facility visits in the past 12 months, far fewer than other studies have documented for women of similar ages.^26^^,^^27^ Therefore, there may be relatively fewer opportunities to reach men at health facilities as compared with women, and every opportunity should be taken to engage men.

We found extensive missed opportunities to reach men who were already engaged with the health system. Among men in need of HIV testing who had attended a health facility, only 7% were offered HIV testing. Other research suggests that similar missed opportunities also exist for other routine screening services.^17^^,^^18^ Given the multiple barriers to testing (such as long wait times, lack of privacy and unfriendly staff),^27^^–^^29^ men may benefit from being offered testing during every facility visit, including at outpatient departments and during guardian visits. Being offered testing may substantially increase overall testing coverage. Of all the men in our sample, 85% of those offered testing accepted, which is similar to other studies in the region.^30^

We found that men primarily attended outpatient departments (for both client and guardian visits). This finding was especially true for men in need of testing where 93% of those who visited a health facility had visited an outpatient department. This result is in line with other research that find that few routine, universal health services are recommended for men who are not ill.^31^ In Malawi, the only recommended preventive service for men younger than 45 years is annual HIV testing.^27^ Outpatient departments should be a key place for engaging men in a range of health services and preventive care interventions.

A large proportion of men’s facility visits were as guardians, where men supported the health care of others. Deliberate efforts to offer screening services to male guardians should be prioritized. Our findings differ from other studies that report that men are not involved in the health care of their families,^32^ although recent literature recognizes that traditional gender roles for caregiving are changing.^33^^–^^35^ Additional research is needed to understand men as guardians and how guardian visits can be best used as an entry point for men’s own health.

How can interventions best capitalize on men’s frequent facility visits? Opt-out services are key to improved uptake of a range of health services.^36^^–^^38^ Screening services could be offered in outpatient department waiting areas while men wait for acute care, thus taking advantage of time already spent at the facility. Self-testing in outpatient department settings can also improve efficiency and minimize the human resources required to administer tests.^39^^,^^40^ HIV self-testing at health facilities has been shown to reduce staff time required for testing, increase testing coverage and is acceptable among men.^22^^,^^41^ Investment in adequate infrastructure and staffing may also be needed for longer-term solutions for screening efforts in outpatient departments.

Our study has several limitations. First, we used HIV testing as a case study to understand the potential reach of facility-based screening services among men. Missed opportunities for facility-based services may be higher for less prioritized health concerns, such as tuberculosis and hypertension. Second, our findings may not be generalizable outside Malawi as health-care-seeking behaviour may differ in countries with higher rates of formal employment and health insurance coverage, such as South Africa. Third, survey data rely on self-reporting and may be susceptible to social desirability bias if men believe they should engage in health services or HIV testing specifically. Fourth, we may have underestimated the proportion of men in need of HIV testing because we did not account for risk factors that increase the recommended frequency of testing – such as known HIV exposure or seeking services for sexually transmitted infections. Finally, our sampling frame did not account for variation in village size or the number of men within each age category in the general population. Sensitivity analyses assigned weights to adjust for these potential biases and found no differences in study results.

Contrary to common beliefs about men, the majority of Malawian men made a health facility visit in the past 12 months, with outpatient departments as the primary entry point for both clients and guardians. Despite frequent facility visits, men were rarely offered HIV testing services, highlighting missed opportunities to engage men already present at health facilities. Increased coverage of routine screening services at outpatient departments and for male guardians could improve programmatic efficiencies by taking advantage of men’s presence at health facilities. 
